# Resilient Health and the Healthcare System. A Few Introductory Remarks in Times of the COVID-19 Pandemic

**DOI:** 10.3390/ijerph19063603

**Published:** 2022-03-18

**Authors:** Anna Augustynowicz, Janusz Opolski, Michał Waszkiewicz

**Affiliations:** 1 Department of Economics of Health and Medical Law, Medical University of Warsaw, 02-091 Warsaw, Poland; 2 Faculty of Engineering and Management, University of Ecology and Management, 00-792 Warsaw, Poland; janusz.opolski@gmail.com; 3 Centre of Postgraduate Medical Education of Warsaw, School of Public Health, 01-826 Warsaw, Poland; mwaszkiewicz@cmkp.edu.pl

**Keywords:** resilience, health system, COVID-19, safety, patient

## Abstract

People across the globe are facing increasingly complex public health emergencies that are responsible for the loss of life, economic and social problems with unprecedented damage and costs. For some sociologists, our society is even “a risk society” and our time is highly violative. Emergencies of different origin: stemming from natural environmental disasters, such as floods, hurricanes, intense drought, technical accidents, social unrest and last but not least—outbreaks of infectious diseases. This decade started with one of the most significant pandemics in the history of man-kind—COVID-19. Hence, the problems of resilient health and healthcare systems have become urgent. Especially since SARS-CoV-2 may cause long-term health threats and recurrent crises. It is very important to have a common language. So far, definitions and concepts of health and healthcare resilience differ substantially and are seldom clearly defined. The aim of this paper is to describe how health and healthcare system resilience is defined to either uncover, recall, or in combination, its concept and prepare an introductory conceptual review as a preliminary step for further studies.

## 1. Introduction

Coronaviruses were neglected for years as human pathogens because they are associated with the relatively mild, self-limiting common cold [1]. The beginning of the 21st century gave rise to the epidemic of highly infectious severe acute respiratory syndromes. 

COVID-19 has resulted in many infected people. A relatively high mortality and changes in the social, cultural and economic spheres can erode and even destroy any development gains. Rather simple replications and mutations of SARS-CoV-2 may cause long-term health threats and recurrent crises. However, the rapid accumulation of data and knowledge development provide fantastic opportunities for developing science-guided policies and procedures for the future prevention and control of outbreaks [1,2,3].

Some countries have done better than others in dealing with the pandemic, so far. Francis Fukuyama said that it is not a matter of the political regime type. The factors responsible for successful pandemic response have been state capacity, social trust and leadership. Countries with a competent state apparatus, a government that citizens trust and listen to and leaders—have performed impressively, limiting the damage they have suffered [4].

Today, reducing the risk of disasters and making social entities resilient is the utmost important aim for countries, communities and individuals. The health sector plays a significant role in this respect.

There is also wide consensus that the global community has to help build more resilient health systems. However, there is no common description of it. Hence, clarifying the meaning could help establish a shared understanding of the concept and build better cooperation among stakeholders.

This paper describes some aspects of resilience, mostly definition and scope issues, in order to either uncover, to recall, or in combination, the concept and improve conceptual knowledge for further study in the Polish health and healthcare system.

Free access to scientific and grey literature published between 1 January 2020 and 31 July 2021 was reviewed. The search was performed in August 2021 in electronic databases: Pub-Med, Scopus, Academia.edu and the Web of Science. The searched keywords were: resilience and health care, resilience and healthcare system resilience and the health system. No formal quality assessment was introduced, but the inclusion of documents was restricted to peer-review papers, official reports and conference documents. A few documents concerning COVID-19 were used in the introduction. This method was a simplification of the five-stage methodological process as outlined in Arksey and O’Malley’s paper [5].

## 2. Resilience

Many multiple terms can be found in literature and practice when speaking of resilience. It means different things for different people: politicians, scholars and practitioners. Anyway, as a concept it covers a wide range of applications in practice, from the gas and oil industry [6] to music ensembles [7].

The word resilience originates from Latin prefix re and the verb salire (to jump, spring, leap back, rebound).

In the English language, the first definition derives from Francis Bacon as the action or an act of rebounding or springing back, rebound, recoil. In the 18th century the “mechanical” usage of the term began. In engineering science and practice, resilience meant elasticity. At the same time, accordingly, the figurative use of resilience began as the quality or fact of being able to recover quickly or easily from, or resist being affected by, something terrible, robustness, adaptability [8].

The real popularity of this notion has exploded in practical, academic and discourse during the last forty–fifty years. Nowadays the concept of resilience is known in physics, engineering, disaster management, psychology, sociology, anthropology, geography and last but not least, public health. The past two decades have accumulated evidence for the interlinkage between human health, biodiversity, the ecosystem and agriculture. Hence, the concept of a social or socio-ecological system is discussed.

Lastly, in the face of COVID-19 and with billions of dollars invested globally to save the economy, resilience was a matter of politics and a policy priority.

The contemporary concept of resilience has evolved from ecology. The Canadian scientist, a visionary of change in nature and society, Stanley “Buzz” Holling, in 1973, defined the term resilience for the ecosystem as a measure of the ability of the ecosystem to absorb changes and still persist. He also considers the stability of a system and compares it with resilience [9,10].

Stability and resilience are important properties of any ecological system and are mutually interdependent, thus a system can be very resistant but still fluctuate greatly [11]. Since then, the ecosystem has been treated as a dynamic entity with the possibility of continuing to work (persist) despite unfortunate changes.

Resilience is used by engineering and material science as the ability of a material to absorb energy when deformed elastically and return to its original state when unloaded [12]. In this mechanical sense, it is the quality of stored strain energy and deflects elastically under load without breaking or being deformed [13].

Resilience has been studied in psychology for decades, but a lack of consensus and plenty of definitions still exist. What is common is that resilience describes the ability to bounce back or overcome some form of adversity and, thus, experience positive outcomes despite an aversive event or situation [14].

Scientists discussing the results of exposure to different traumatic situations and resilience have concluded that it is a complex construct. It may be defined differently in individuals, families, organizations, societies and cultures. The proposed definition included a stable trajectory of healthy functioning after a highly adverse event, a conscious effort to move forward correctly as a result of a lesson learned, the capacity of a dynamic system to adapt and a process to harness resources to sustain well-being [15].

Resilience is also an issue of increasing importance for city managers and policymakers. Since the turn of the millennium, many big cities are increasingly adopting resilience strategies to plan for and manage possible risk of different origins [16]. Urban resilience refers to the ability of an urban system—and all its constituent socio-ecological and socio-technical networks across temporal and spatial scales—to adapt to many changes; to maintain or rapidly return to desired functions in the face of disturbances, adapt to change and quickly transform systems that limit current or future adaptive capacity [17].

At the beginning of the 21st century spatial resiliency emerges to refer to the importance of ecological legacies (e.g., species or habitat characteristics that persist after disturbances and provide “ecological memory” during organization and connectivity among neighboring systems for withstanding disturbances and avoiding regime shifts in a broader spatial extent than individual focal systems).

Changing societal views of nature and the recognition of the dynamics of ecological and social systems form the basis for the emerging resilience of different ecosystems within the context of the importance of wildlife management [18,19].

Carpenter et al., in his well-known paper [20], simply asked “Resilience of what to what?”. Carpenter’s “to what” means variables that influence system performance and may change unexpectedly over the system’s lifetime.

Wied, Oehman and Welo [12] propose the third category “how”. A set of variables mediating between uncertain conditions and system performance.

So-called organizational resilience has been defined as “the maintenance of positive adjustment under challenging conditions such that the organization emerges from those conditions strengthened and more resourceful” [21,22].

Resilience is sometimes divided into planned resilience and an adaptive one [23]. Planned resilience includes, among others, pre-disaster activity, e.g., plans to avoid or minimize the effect of a crisis throughout business continuity and risk management. Adaptive resilience emerges during the post-disaster period as organizations develop new capacities by responding to emergent situations.

There are three possibilities regarding resilience as a meta-concept: an outcome, as a process and capabilities. Resilience as a process has three phases (periods): before the onset of disaster, within disaster and after it. The first is devoted to preparing against a disaster; the second is a response to a situation and learning from experiences; and the third one is nothing else but a cognitive, behavioral, managerial, financial resource and special activity to handle the impact of disaster [24].

Stephanie Duchek pointed out very clearly in her excellent paper [24] that to survive in an uncertain environment and foster future success, the organization must be able to handle all these manifestations of the unexpected. Resilience differs from related constructs as flexibility, agility or robustness. They have almost identical elements but resilience deals with unforeseen threats and crises, not daily problems. Furthermore, resilience includes an adaptation aspect that could allow the company to come out of a crisis even stronger than before.

A significant strand of literature is aimed at the community level. In particular, resilience has emerged as an attractive perspective regarding “acute” public health situations stemming from health disasters, e.g., sudden, large-scale, usually chaotic events of an acute onset resulting in significant psychological, physical, social, economic and environmental harm [25,26].

The UN Office for Disaster Risk Reduction defines resilience as the ability of a system, community or society exposed to hazards to resist, absorb, accommodate to and recover from the effects of a threat in a timely and efficient manner, including through the preservation and restoration of its basic structures and functions [27].

In recent years, many governments, after the evaluation of so-called critical infrastructure, came to the conclusion that such institutions should maintain functions following disaster, crisis and so on. American scientists have developed a comprehensive resilience assessment framework for evaluating the resilience of infrastructure and economic systems. However, it is of general value and can be used in other sectors. Increasing resilience involves three capabilities: absorptive, adaptive and restorative capacities. The system is able to absorb the impact of a crisis, adjust to the undesirable condition and finally rapidly return to the “status que ante” or even better organization [28].

## 3. Activity for Health Systems

In the World Health Report 2000, it was said that “in today’s complex world it can be difficult to say exactly what a health system is. What it consists of, and where it begins and ends. Nevertheless, the Report defines“ a health system to include all the activities with the primary purpose to promote, restore or maintain health [29].

This definition provides a clear conceptual understanding and comprehensive framework by addressing five conceptual questions: (1) What are the boundaries? (2) What is the system for? (3) What architecture? (4) How good is the system in terms of its performance? (4) How can we relate system architecture to performance [30]?

However, a key debate when considering international frameworks is still the question of where to set the boundaries of health systems and what responsibilities lie within the jurisdiction of the health system [31]. Furthermore, the distinction between the health system and the healthcare system is not precise enough in the literature. Most papers refer to the provision of health services and investment in health services. This is a healthcare system. Health care as an activity or function of caring for people concerning specific, e.g., health visits: promotion, diagnosis, treatment and rehabilitation.

However, the healthcare system is a set of values, knowledge, specialized institutions—including human resources for health—and coordinated activities. All this serves to function for the maintenance and improvement of the health status of individuals and societies through prophylactic measures, the control of diseases and other health conditions which cause pain, suffering and disability.

The health system goes beyond the boundaries of a healthcare system. It encompasses the healthcare system but also organization and activity regarding the social determinants of health.

In the majority of papers there is a misunderstanding in this respect. According to the WHO, the health system has three core objectives: improving the population’s health, responding to people’s expectations and protecting against the cost of ill health. To reach them, it is necessary to fulfill four functions [32]; goals—deliver services, finance them, create resources and stewardship.

These items are presented as six “building blocks”, e.g., governance and leadership, health financing, human resources for health, health information systems, medicine and medical products and service delivery. The building blocks must work together to respond to changing health needs and demands in order to reach the goal of the system: improving the health of the population.

Better health is the raison d’etre of both systems, the health and health care systems—its primary and defining goal. If systems did nothing to protect or improve health, there would be no reason for them to exist.

Previously, five attributes of the health system were usually evaluated: quality, efficiency, equity, accountability and sustainability [33]. Today, resilience is important as the sixth one [34].

## 4. Health System Resilience

As the COVID-19 pandemic ravages countries, health systems have mainly been ill-prepared to tackle such an unprecedented burden. The health systems were not resilient. The health systems do not have the resilience. “Resilient” is a feature of the system; “resilience” makes the system resilient.

Many understandings are used worldwide when speaking of what health system resilience is and what this term conveys.

In Communication from the Commission on effective, accessible and resilient systems, resilience means “adapting effectively to changing environments and identifying and applying solutions to tackle significant challenges…” [34].

In the WHO’s document of the Western Pacific Region [35] resilient health systems are able to cope with internal and external shocks and recover quickly and continue to prepare for and adapt to changing environments. To ensure resilience for combating shocks and sustaining progress, Member States need to enhance public health preparedness, develop community capacity for health protection and promotion and provide adaptability and sustainability to the health system.

Margaret E. Kruk et al. define health system resilience as the capacity of health actors, institutions and populations to prepare for and effectively respond to crises; maintaining core functions when a crisis hits and informed by lessons learned during the crisis, reorganize conditions required [36]. Resilience requires planning and investment in “slow variables”, e.g., HRH, the information system and “fast variables”, e.g., protective equipment and isolation wards. It requires the systematic building of collaboration and trust with communities ahead of a crisis. Human resources are also important [37].

Margaret Kruk et al. also present a resilience index with five characteristics, fourteen aims, twenty-five measures and twenty-five rationales. It is one of the most important papers on the issue. The authors propose its “operationalization”, e.g., what should be done and what a system should have in the case of building resilience. 

For Karl Blanchet’s resilient healthcare system, it absorbs the shock of such emergencies by learning from previous experiences and transforming itself to respond to pandemics and ensure the full provision of other health services [38]. They also propose management capacity for conceptual analysis, namely, knowledge, uncertainties, interdependence and legitimacy. Knowledge is the “capacity to collect, integrate and analyze different forms of knowledge and information”; uncertainties—the “ability to anticipate or develop legitimate institutions and cope with uncertainties and surprises”; interdependence—the “capacity to manage interdependence: to engage effectively with and handle” and legitimacy—the “capacity to build or develop legitimate institutions accepted and contextually adapted”. It seems close to Norris and Biddle’s proposal that resilience is simply a set of capacities [25].

Jennifer B. Nuzzo [39], in literature, has found 16 health system resilience attributes, which are summarized as follows: core health service capabilities, barriers to healthcare access, maintaining critical infrastructure and transportation coordination and partnership, communication, flexible plans and management structure, legal preparations, surge capacity, altered standard of care, health workforce, medical supplies, infections prevention and control, commitment to quality improvement, plans for post-event recovery.

Victoria Haldane et al. analyzed 28 countries based on a purposive selection, including positive and negative outliers concerning reported COVID-19 death [40]. The similarities across countries with divergent health outcomes make it evident that there is no one silver bullet constituting a resilient health system. However, four characteristics have been common in countries with low mortality. 

Resilient health systems are systems that: (1) activate a comprehensive response which consists of responses that consider and address health and well-being as intertwined with social and economic considerations; (2) adopt capacity within and beyond the health system to meet the needs of communities; (3) preserves functions and resources within and beyond the health system to maintain pandemic-related and non-related routine and acute care and (4) reduce vulnerability to catastrophic losses in communities in terms of health, well-being and finances.

The authors underlined that health is more than healthcare. A whole-of-government approach to health and well-being is needed to create a collective healthy population able to prevent and respond to crises.

The German National Academy of Science and Engineering issued the document on the resilience and performance of healthcare systems in times of crisis [41]. Here, the term resilience is the ability to prepare for and cope with sudden and hard to foresee adverse events (shocks) and use the lessons learned to adapt and improve the relevant systems. Resilience, as a process, can be broken down into five phases: prepare, prevent, protect, respond and recover and re-imagine. Measures to strengthen healthcare systems exist in several different areas. These include information and communication, the structure of the healthcare system and its strategic reserves, the relationship between relevant institutions.

Close to the above proposition is a definition elaborated by the EU Expert Group on Health System Performance. The authors insist that the definition encompasses the core features of resilience as they describe in several conceptual papers and also include relevant surveys from non-health disciplines. According to them: “Health system resilience describes the capacity of health to proactively foresee, absorb and adopt to shocks and structural changes in a way that allows it to sustain required operations, resume optimal performance as quickly as possible, transform its structure and functions to strengthen the system and possibly reduce its vulnerability to similar shocks and structural changes in the future.” [42].

A prevailing number of documents, papers and surveys deal with resilience as a tool against disasters, shocks and so on. In other words, “with a sudden and extreme change which impacts a health system and is thus different from the predictable and enduring health system stresses” [43].

However, there are opinions, within a so called “wider vision of resilience” implying that the relevance of resilience, from a health system perspective, goes beyond sudden crisis when considering the myriad of changes that can affect the system in a more insidious way [44].

In light of these reflections, health system resilience is rather highly confused. The concept is still ambiguous as such and depends on a particular discipline or even personal perception. Various conceptual frameworks used are not commonly accepted. Hence, it is difficult to measure and understand the real world in circumstances without clear boundaries and bad communication due to a lack of common language.

It is necessary to solve this problem and reconsider the whole issue starting with the discussion on notions and definitions. All definitions differ in wording, but most of them continue to use one or two words: ability and capacity. It could be a good trigger to start elaborating on a relevant definition.

## 5. Conclusions

1. Resilience is crucial for the proper functioning of health institutions and health systems.
2. The first step to having resilient health and healthcare systems is common languages, e.g., a common understanding of the scope and definition: what resilience is; its features, range and boundaries, to whom it is organizing, how is it built and how does it function.
3. Definitions and concepts of health and healthcare resilience differ substantially. Moreover, they are seldomly clearly defined.
4. All definitions of resilience have a common core: the capacity or ability to cope with a disaster, handle it and reconstruct the system, if relevant.
5. In many papers published after the onset of COVID-19, the problem of maintaining the routine activity of health institutions is underlying.
6. Maintaining resilient health and a healthcare systems is the overarching responsibility of governments: both central and local.
7. Further studies of different aspects of resilience regarding the Polish health system are noteworthy.

## Data Availability

All data are available from the corresponding author.
